# Prevention of advanced colorectal cancer by screening using the immunochemical faecal occult blood test: a case–control study

**DOI:** 10.1038/sj.bjc.6601002

**Published:** 2003-07-01

**Authors:** M Nakajima, H Saito, Y Soma, T Sobue, M Tanaka, A Munakata

**Affiliations:** 1 First Department of Internal Medicine, Hirosaki University School of Medicine, 5 Zaifu-cho, Hirosaki 036-8562, Japan; 2 Centre for Education and Research of Lifelong Learning, Hirosaki University, 1 Bunkyo-cho, Hirosaki 036-8560, Japan; 3 Cancer Information and Epidemiology Division, National Cancer Center Research Institute 5-1-1, Tsukiji, Chuo-Ku, Tokyo 104-0045, Japan; 4 Department of Pathology, Hirosaki University School of Medicine, 5 Zaifu-cho, Hirosaki 036-8562, Japan

**Keywords:** colorectal cancer, screening, advanced cancer, immunochemical test, faecal occult blood, case–control study

## Abstract

To evaluate colorectal cancer screening with faecal occult blood testing (FOBT) in terms of prevention of advanced cancers, we conducted a case–control study in the areas where an annual screening programme with immunochemical FOBT has been offered to all inhabitants aged 40 years or over. Cases were 357 consecutive patients in the study areas clinically diagnosed as having advanced colorectal cancer or a tumour invading the muscularis propriae or deeper, that is, T_2_–T_4_ in TNM classification. Three controls were selected for each case matched by gender, age, residential area and exposure status to screening within 1 year before case diagnosis. The odds ratios (ORs) of developing advanced cancer were calculated using conditional logistic regression analyses. The OR for those screened within 3 years before the diagnosis *vs* those not screened was 0.54 (95% confidence interval (CI) 0.29–0.99). The ORs were lower for rectum than for colon (0.32–0.73 and 0.84–1.18 for rectum and colon, respectively). For those screened within the past 3 years, OR of developing advanced cancer in the rectum was 0.32 (95%CI: 0.12–0.84). A screening programme with immunochemical FOBT can be effective for prevention of advanced colorectal cancer. Risk reduction appears to be larger for rectal than for colon cancer.

Colorectal cancer is one of the highest causes of cancer deaths in many countries (Schottenfeld and Winawer, 1996). Accordingly, prevention is an issue of global importance. Although colorectal cancer is considered to be closely related to environmental factors such as food and life style, effective methods for primary prevention have yet to be established (Schottenfeld and Winawer, 1996; Rhodes, 2000). Under these circumstances, secondary prevention by early detection is a practical way to lessen the burden. Among several modalities that have been proposed for colorectal cancer screening, the faecal occult blood test (FOBT) or the Haemoccult test has been demonstrated by three randomised controlled trials to reduce mortality from colorectal cancer by 15–33% with annual or biennial screening (Mandel *et al*, 1993,^1999^; Hardcastle *et al*, 1996; Kronborg *et al*, 1996). It is, therefore, generally considered that the effectiveness for FOBT has already been established.

In Japan, screening programmes with various FOBTs by immunochemical method have been performed. Among the screening programmes with those FOBTs, efficacy of the screening by the immunochemical haemagglutination test or the immunochemical FOBT was consistently suggested by several studies, although they were observational (Hiwatashi *et al*, 1993; Saito *et al*, 1995,^2000^; Wada *et al*, 1996; Zappa *et al*, 1997). Screening with this type of FOBT is also considered to be effective in terms of mortality reduction (Young *et al*, 2002).

The ultimate gains derived from a screening programme are reductions of serious illness, as well as death, among the people screened (Morrison, 1992). Reduction of the incidence of advanced cancer is crucial in terms of quality of life. The immunochemical FOBT (Saito *et al*, 1984; Saito, 1996), which is widely used as a screening test in Japan, has been shown to be superior to the Haemoccult test in sensitivity, with similar specificity, as reported both in known colorectal cancer (St John *et al*, 1993), and in asymptomatic populations (Iwase 1992; Petrelli *et al*, 1994; Allison *et al*, 1996; Castiglione *et al*, 1996; Saito and Yoshida, 1996). Furthermore, sensitivity of the immunochemical FOBT is higher for adenomas (St John *et al*, 1993), intramucosal cancer (Iwase 1992; Saito and Yoshida, 1996) and Dukes A cancer (Castiglione *et al*, 1996). On the basis of these reports, it is expected that screening with the immunochemical FOBT provides larger effect on the incidence reduction of advanced colorectal cancer as compared to that with the Haemoccult test. This assumption might be supported by a report that risk of developing an interval cancer after the immunochemical FOBT is significantly lower than that after the Haemoccult test (rate ratio=2.64: Zappa *et al*, 2001).

The purpose of this study is to evaluate whether screening with the immunochemical FOBT reduces the incidence of advanced colorectal cancer, which requires surgery. Therefore, in the present paper, we conducted a case–control study to evaluate whether screening with the immunochemical FOBT reduces the risk of developing advanced colorectal cancer in the area where annual screening with the immunochemical FOBT has been performed.

## MATERIAL AND METHODS

### Study district and screening programme

Colorectal cancer screening has been conducted using a one-day immunochemical FOBT test (Imudia-Hem Sp: Fujirebio, Tokyo, Japan) since 1986 in Aomori Prefecture, Japan. Details of the study district and the screening programme were described in a previous paper (Saito *et al*, 1995). The district consists of two small cities, 13 towns and 11 villages. The entire population of the screening areas is 276 819 in 1992. Screening was annually offered to all men and women, who held national health insurance, aged 40 years or over (101 136 persons in 1992) through the Aomori Screening Centre for Cancer and an average of 16 000 persons participated in the screening each year before 1992 (Saito *et al*, 1995). Accordingly, the participation rate was approximately 16%. Nearly 60% of the participants were female subjects. The study area had not had colorectal cancer screening prior to 1986, and a complete listing of residents had been conducted just before the programme was started. The immunochemical FOBT test was performed according to the original method (Saito *et al*, 1984; Saito, 1996) at the laboratory in the Centre and records of all screening examinations were maintained. A positivity rate for the FOBT was 2.4% (Saito *et al*, 1995). Screenees who tested positive for FOBTs were recommended to undergo diagnostic investigation by sigmoidoscopy with double-contrast barium enema or by total colonoscopy at hospitals in or near the study area. Sigmoidoscopy in combination with barium enema was more prevalent than colonoscopy as a diagnostic examination in this programme, that is, 70% or more before 1992. There were 66 cases of colorectal cancer found in the screening programme during 1989–92. Screenees, who were found to have colorectal cancers or adenomas, were treated by surgical or endoscopic resection. The Centre retrieved detailed information on diagnosis and treatment from the hospitals at which diagnostic investigation and treatment were performed.

### Definition and selection of cases and controls

Cases were defined as the consecutive patients clinically diagnosed as having advanced colorectal cancer that invaded to the muscularis propriae or deeper, which required surgery, that is, T2–T4 in TNM Clinical Classification or A–C in the original Dukes classification (Sobin and Wittekind, 1997). Among Dukes A cancers, those cancers limited to submucosal layer were not included in advanced cancer cases in this study. Diagnoses of cases were performed in the study areas from 1 April 1989 to 31 December 1992. For inclusion, case subjects were 40 years or older at the time of diagnosis and had been living in the same area since 1986 when the screening programme was started. Patients with a previous history of colorectal cancer were excluded. Potential case subjects were obtained from the files of the cancer registries that cover the whole prefecture. Diagnoses of colorectal cancers were scrutinised by reviewing medical records for colonoscopic and/or radiographical findings, anatomic location of the cancer, histological diagnosis and depth of cancer invasion.

There were 543 potential case subjects clinically diagnosed during the period. Confirmation of diagnosis, site and stage of the cancer was made in 423 subjects by reviewing medical records. A total of 12 were excluded because they were less than 40 years old at the time of diagnosis. We further excluded 54 cases of early cancer whose cancer did not invade muscularis propria. More than half of these 54 cancers were those confined to the mucosa, but we could not determine in some cases with or without cancer extension through muscularis mucosae into the submucosa. In total, there were 357 cases of clinically diagnosed advanced colorectal cancers that met the above criteria. All the cases were identified in the list of residents of the study area. These cases included 62 fatalities documented in a previous case–control study (Saito *et al*, 1995). To minimise the influence of selection bias, they were included in the case subjects. Among the total of 357 cases, there were six who had screening tests within 1 year before their diagnoses. These six had negative FOBT. Further, among 66 screen-detected cancer subjects, there were 33 with advanced colorectal cancer that also met the above criteria, thus making them subjects for additional analysis.

For each case, we randomly selected three controls from the list of residents in 1986 in the study area, where the corresponding case lived, matched to cases by year of birth (±3 years), gender and residential area within the town or village. According to a matching criterion for a case–control study that evaluates screening efficacy in terms of risk of invasive disease (Sasco *et al*, 1986), controls were matched to cases also by exposure status just before case diagnoses. For the 351 cases who did not have screening tests within 1 year before their diagnoses, controls were required not to have screening tests during the same period. There were 1047 controls eligible for the above criteria. For the other six cases, who had screening tests within 1 year before their diagnoses, and each of 33 screen-detected cases, controls were selected from among those who participated in the screening programme in the same year as the case diagnosis. Each control subject had to be alive when the case subject was diagnosed. Control subjects, with a history of colorectal cancer before the screening programme was started in 1986 or 1987, were excluded. Of 1164 controls selected for 390 cases, 1071 had the same birth year as those of the corresponding cases. We did not encounter any control subjects who developed colorectal cancer during the period from 1986 until the time of case diagnosis.

### Comparison of screening history and analyses

Screening histories were investigated by reviewing the records of the screenees by staff of the Centre who were blind to whether the subjects were cases or controls. Screening histories of cases and controls were reviewed within the same calendar period of 5 years before case diagnosis. Conditional logistic regression analyses were performed in the same manner as previously reported using PROC PHREG with the SAS computer programme (Saito *et al*, 1995). The odds ratios (ORs) of developing advanced colorectal cancer were calculated primarily using the above 357 case–control sets. As screening exposure within 1 year was matched between cases and controls, it was not used for case–control comparison. Odds ratios were calculated for those having at least one screening test within 2, 3, 4 and 5 years before case diagnosis *vs* those having no screening test in the corresponding periods. To investigate the optimal screening interval, ORs were also calculated for those having their most recent screening history in each of the 2–5 years before the date of diagnosis *vs* those not screened in that year segment. With 390 case–control sets including those of 33 screen-detected cancers and their controls, ORs were also calculated in the same manner as in the above 357 sets for those having been screened within 2–5 years before case diagnoses. Statistical significance was evaluated at a 5% level, and 95% confidence intervals (CI) were presented for estimated OR.

## RESULTS

The age, stage and site distributions of the 357 clinically diagnosed case subjects are shown in Table 1

**Table 1:** Clinical characteristics of case subjects with clinically diagnosed and screen-detected advanced colorectal cancer

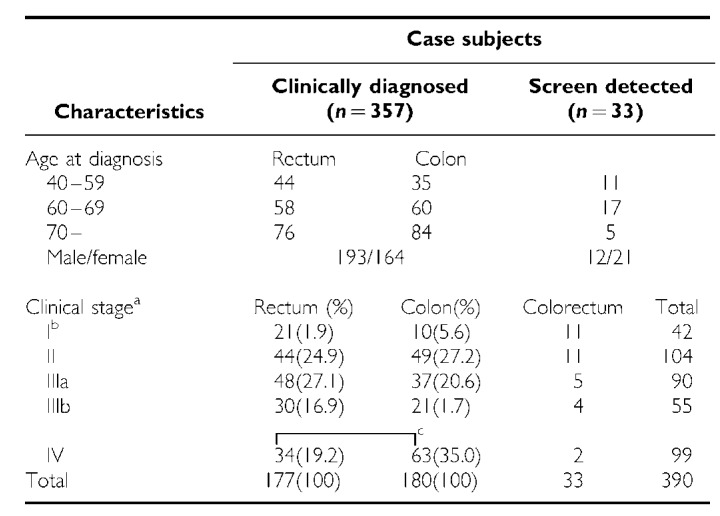	

Advanced cancer was defined as cancer invaded to the muscularis propriae or deeper.

^a^Clinical stages were based on the classification proposed by ‘Japanese Society for Cancer of the Colon and Rectum’ (1995). The stages are mainly defined by depth of the primary tumour; Stage 0: within the mucosa; Stage I : invading the submucosa or muscularis propriae; Stage II : beyond the muscularis propriae, but not directly invading into the other organs; Stages III and IV: directly invading into the other organs. Regardless of tumour depth, however, metastasis to the first group, the second or third group and the fourth group lymph nodes indicates Stage IIIa, Stage IIIb and Stage IV, respectively. Stage IV can be diagnosed by existence of peritoneal or distant metastasis.

^b^Case subjects with tumour invasion limiting to the submucosa were excluded from the present study.

^c^*P*<0.05 (by *χ*^2^ test).

. Clinical stages were shown based on the classification of the Japanese Society for Cancer of the Colon and Rectum (1998). About half of the case subjects had rectal cancers. The ratio of colon to rectal cancers was higher for those aged 70 years or over than that for those of younger ages although not statistically significant. The proportion of stage IV cancers was higher with colon (35.0%) than with rectal lesions (19.2%) with a statistical significance (*P*<0.05 by *χ*^2^ test, Table 1) and was similar among the age groups. The stage distributions of screen-detected case subjects are also shown in Table 1.

Odds ratios of developing advanced colorectal cancer were calculated for individuals having screening histories within 2–5 years before case diagnosis, as compared with those not screened, using 357 case–control sets. Risk of developing advanced colorectal cancer was reduced by 28–46% among individuals having at least one screening within 2–4 years before case diagnosis, with statistical significance for those screened during the past 3 years (Table 2

**Table 2:** Odds ratios (ORs) of developing advanced colorectal cancer in individuals screened within 2,3,4 and 5 years before case diagnoses

	**Number of subjects available^a^**		**Number of subjects screened (%)^b^**		
**Years before diagnosis**	**Cases**	**Controls**	**Cases**	**Controls**	**ORs (95% CI)**
Within					
2 years	357	1065	10 (2.8%)^c^	47 (4.4%)^c^	0.60 (0.29–1.23)
3	349	1040	14 (4.0)	72 (6.9)	0.54 (0.30–0.99)
4	311	931	23 (7.4)	92 (9.9)	0.72 (0.44–1.17)
5	218	649	24 (11.0)	74 (11.4)	0.96 (0.57–1.59)

a The number of subjects who had the chance to participate in screening during each period.

b Subjects with screening histories/number of subjects (%). Odds ratios were calculated for previous history within 2, 3, 4 and 5 years before case was diagnosed as having advanced cancer compared with no screening history in those intervals, using conditional logistic regression analysis.

c Number of subjects who had screening histories within 1 year before case diagnosis was not included, as screening exposure status within 1 year of case diagnosis was matched between cases and controls.

ORs within 1 year are equal to that of within 2 year.

). The ORs calculated for the 357 case–control sets by number of years since the most recent screening were 0.60 (95% CI: 0.29–1.23) after 2 years and 0.58 (95% CI: 0.22–1.52) after 3 years following the most recent screening, but no reduction in risk was observed after more than 3 years. When ORs were calculated including 33 screen-detected cancers and their controls, the values within 2 and 3 years were closer to unity than those from 357 case–control sets (Table 3

**Table 3:** Odds ratios (ORs) of developing advanced colorectal cancer for those screened within 2, 3, 4 and 5 years before case diagnoses when analysed after inclusion of 33 screen-detected cancers and their controls

	**Number of subjects available^a^**		**Number of subjects screened(%)^b^**		
**Years before diagnosis**	**Cases**	**Controls**	**Cases**	**Controls**	**ORs (95%CI)**
Within					
2 years	390	1164	26 (6.7%)	94 (8.1%)	0.76 (0.45–1.28)
3	382	1139	34 (8.9)	129 (11.3)	0.69 (0.43–1.10)
4	343	1027	42 (12.2)	155 (15.1)	0.72 (0.47–1.11)
5	243	724	42 (17.3)	127 (17.5)	0.98 (0.62–1.55)

a The number of subjects who had the chance to participate in screening during each period.

b Subjects with screening histories/number of subjects (%). Odds ratios were calculated for previous history within 2, 3, 4 and 5 years before case was diagnosed as having advanced cancer compared with no screening history in those intervals, using conditional logistic regression analysis.

). We performed the following analyses using 357 case–control sets.

Odds ratios of developing advanced colorectal cancer were also calculated according to the subgroups of gender, age, stage and anatomic location of the cancer (Tables 4

**Table 4:** Odds ratios (ORs) of developing advanced colorectal cancer for those screened within 2, 3, 4 and 5 year before diagnosis, in the subgroups according to gender and age

	**ORs^a^ (95%CI)**	
**Years before diagnosis**	**Gender (male/female) (*n*=193/164)**	**Age (40–69 years/≧70 years) (*n*=197/160)**
Within		
2 years	0.63 (0.23–1.72)/0.58 (0.21–1.59)	0.53 (0.22–1.25)/0.82 (0.23–2.93)
3	0.66 (0.29–1.47)/0.44 (0.18–1.08)	0.47 (0.22–1.00)/0.71 (0.26–1.90)
4	0.78 (0.38–1.59)/0.66 (0.34–1.31)	0.62 (0.33–1.16)/0.92 (0.41–2.06)
5	0.99 (0.47–2.10)/0.93 (0.47–1.86)	0.72 (0.36–1.46)/1.36 (0.64–2.88)

a Odds ratios calculated for previous history within 2, 3, 4 and 5 years before diagnosis, compared with no screening history in those intervals, using conditional logistic regression analyses. Separate model is used to calculate OR in each line.

and 5

**Table 5:** Odds ratios (ORs) of developing advanced colorectal cancer for those screened within 2, 3, 4 and 5 year before diagnosis, in the subgroups according to clinical stage and anatomical location.

	**ORs^a^ (95%CI)**	
**Years before Diagnosis**	**Stage (I–IIIb/IV) (*n*=260/97)**	**Location (rectum/colon) (*n*=177/180)**
Within		
2 years	0.52 (0.22–1.22)/0.90 (0.24–3.36)	0.40 (0.13–1.20)/0.88(0.34–2.26)
3	0.55 (0.28–1.08)/0.53 (0.15–1.87)	0.32 (0.12–0.84)/0.84(0.39–1.81)
4	0.61 (0.34–1.10)/1.10(0.43–2.80)	0.43 (0.19–0.97)/1.06(0.57–2.00)
5	1.01 (0.56–1.81)/0.81(0.28–2.34)	0.73 (0.32–1.68)/1.18(0.61–2.27)

a Odds ratios calculated for previous history within 2, 3, 4 and 5 years before diagnosis, compared with no screening history in those intervals, using conditional logistic regression analyses. Separate model is used to calculate OR in each line.

). Similar ORs were obtained for males and females. Odds ratios were higher for those aged 70 years or over than those aged 69 years or younger (Table 4). There was no significant difference between ORs for overall cancers and stages I–IIIb (Tables 2 and 5). Regarding anatomic location, the ORs of developing advanced cancer for those having at least one screening history within 2–5 years were higher for the colon than for the rectum with statistical significance for the OR of developing rectal cancer for those having screening tests during the past 3 and 4 years (Table 5).

We further calculated ORs for rectal cancers and colon cancers using stages I–IIIb cancers, because the rates of stage IV cancers were significantly different between the colon and rectum (Table 1). Odds ratios for those screened within 2–5 years before case diagnoses were higher for colon (0.76–1.39) than for rectum (0.31–0.74) also after excluding stage IV cancers, albeit without statistical significance.

## DISCUSSION

The present study was intended to evaluate the screening with the immunochemical FOBT in terms of prevention of advanced cancers in the colorectum that require surgery. Several case–control studies have shown screening to provide strong protection against development of advanced cervical cancer (Clark and Anderson, 1979; Dunn and Schweitzer, 1981; Macgregor *et al*, 1985; Van der Graaf *et al*, 1988) and although there have been no randomised controlled trials, the efficacy of cervical cancer screening has therefore been considered to be established. The present case–control study indicates that screening with immunochemical FOBT reduces the risk of developing advanced colorectal cancer. Subjects who had undergone at least one screening test during the previous 4 years had a reduced risk of bearing advanced colorectal cancer by 28–46% as compared to those who were unscreened (Table 2).

In this study, main analyses were performed using 357 clinically diagnosed cancers. This is because, screen-detected and symptom-detected cases should be kept separate in the analysis to avoid mixing prevalence and incidence ORs (Sasco *et al*, 1986). For screen-detected cases one is estimating the OR of prevalence, rather than incidence, rates of disease among various screening histories (Sasco *et al*, 1986). The purpose of this study is to estimate the incidence rate of advanced colorectal cancer among those with screening histories as compared to those without having them. Accordingly, ORs were calculated using clinically diagnosed cases and their controls and then including screen-detected case–control sets. When analyses were performed including 33 screen-detected cases and their controls, ORs within 2 and 3 years became closer to 1.0 than the original ORs (Tables 2 and 3). This might be because, the time at which diagnosis is made shifts forward depending on the distribution of lead times. Controls were matched to cases by exposure status just before case diagnoses. This matching was done because a chance of appearing in the study as cases, which must be equal between a case and controls of a matched set, is different depending on with or without screening exposure just before case diagnoses (Sasco *et al*, 1986). This is straightforward in asymptomatic screen-detected cases and their controls, and it might be the same for symptom-detected cases and controls. In fact, ORs were much lower when screening histories were compared between 357 cases and their controls matched only by sex, age and residential area, that is, 0.18(95% CI: 0.08–0.42), 0.35(0.18–0.70), 0.35(0.20–0.62), 0.48(0.30–0.77) and 0.58(0.36–0.94) for those screened within 1, 2, 3, 4 and 5 years before case diagnoses, respectively. Odds ratios shown in Tables 2 and 3 might be overestimated due to overmatching.

Concerning the degree of efficacy, 60% reduction in risk of death from colorectal cancer was found in the previous study of screening using immunochemical FOBT alone (Saito *et al*, 1995). Hiwatashi *et al* (1993) reported 74% reduction of risk for those screened with Haemoccult or immunochemical FOBT (1993). The present study to investigate the efficacy of early detection suggested 46% reduction in risk of developing advanced cancer. It seems to be reasonable that the magnitude of reduction in risk of advanced cancer is smaller than that for risk of death from cancer, because mortality reduction reflects the sum of prevention of invasive cancers and stage shift of advanced cancers.

A reduction in incidence of colorectal cancer after screening has recently been demonstrated by a randomised controlled trial, although the reduced incidence might be attributable to many colonoscopies performed rather than FOBT screening (Mandel *et al*, 2000). Whichever the contributor is, the incidence reduction would have been achieved through removal of adenomas in the above trial. In this study, incidence reduction of advanced cancer was strongly suggested. The reason for reduction in risk of advanced cancer by screening would be a stage shift of advanced cancers to early cancers or cancers confined within the submucosal layer. There were 33 early cancer cases, which were not included in the cases in this case–control study, among 66 screen-detected cases during 1989–92. Although the proportion of early cancers among clinically diagnosed cases could not be determined due to inability to confirm stages in many subjects, it might be approximately 13%, that is, 54 early cancers among 423 cases reviewed, being much lower than that for screen-detected cases. Another possible reason is presumably the effect of endoscopic polypectomy of adenomas from which colorectal cancers are believed to arise (O'Brien *et al*, 1990; Mandel *et al*, 2000). Screenees having colorectal polyps sized 5 mm or more were primarily treated by endoscopic polypectomy in the present study areas. Therefore, it can be estimated that polypectomy would have been performed much more frequently than in the unscreened group. Although there are no comprehensive data available about polypectomy in screenees, polyps larger than 1 cm in diameter were found in approximately 0.2% of the screenees through the same screening programme in a neighbouring area with similar characteristics to the study area (Saito and Yoshida, 1996). Therefore, it is most likely that reduced risk of advanced cancer after screening is partially due to the effect of polypectomy.

In the subgroup analysis according to ages, ORs were higher for those of 70 years old or over than for those of younger ages (Table 4). A higher proportion of colon cancer to rectal cancer among those aged 70 years or older than those of younger ages might be a reason for this result (see Table 1 and Table 5 and below). It is possible that efficacy of preventing advanced colorectal cancer might be smaller for individuals aged 70 years or over as compared to that for younger ages. However, this needs to be investigated by additional studies. In the subgroup analysis by anatomic location, the ORs of developing advanced cancer for those screened within 2–5 years were higher for the colon (0.84–1.18) than for the rectum (0.32–0.73) (Table 5). This result was similar to that of a previous study, which suggested that the OR of developing fatal cancer with screening within 3 years before case diagnosis was higher for the colon (0.56) than for the rectum (0.39) (Saito *et al*, 1995). Although significantly higher proportion of stage IV cancers was included in subjects with colon cancer than in those with rectal cancer (Table 1), calculated ORs for colon cancer after excluding these were still higher than those for rectal. One plausible explanation is that sensitivity of diagnostic examination is higher for distal than proximal cancers because the recommended modality has been flexible sigmoidoscopy in combination with barium enema. Obviously, barium enema examination is less sensitive in detecting early lesions than endoscopy. Thus, distal lesions are more likely to be detected than proximal lesions. It would be possible that the efficacy of screening differ between rectal and colon cancers. Several studies have reported that haemoglobin loses its immunoreactivity during transit through the colon (Young and St John, 1992; Saito, 1996). Thus, it is possible that sensitivity of immunochemical FOBT may be higher for rectal than colon early lesions despite the finding that sensitivity does not differ between proximal and distal cancers (St John *et al*, 1992,^1993^). Another explanation is that the natural history of cancer may vary between the rectum and the colon. Genetic alterations may be different between cancers in the proximal colon and those in distal sites (Kern *et al*, 1989), so that a growth differential could arise. Further studies are clearly needed to explain the difference in ORs between rectal and colon cancers.

Some potential confounding factors should be considered with observational studies such as the present case–control investigation (Cole and Morrison, 1980). Diagnoses of cases, which were here verified by reviewing the medical records, should be accurate. Since the screening histories for cases and controls were based on the same data source, recall bias could be eliminated. In addition, confounding by effects of previous screening could be excluded because our case and control subjects had not been screened before starting the screening programme with immunochemical FOBT in 1986. Further, the screening histories of each case and the corresponding controls were evaluated for the same time period and in exactly the same way. For that reason, differences in the time frame for collection of cases and controls did not bias exposure opportunity.

The main defect of case–control studies in the evaluation of the efficacy of screening is self-selection bias (Cole and Morrison, 1980). It is generally considered that risk of the disease is different between individuals who are willing to be screened and those who are not. Accordingly, the efficacy of the screening might be overestimated. However, in this study, ORs increased with the extension of duration after the last screening and values were 1.0 or higher for those having their most recent screening test 4–5 years before diagnosis (Table 3), suggesting that self-selection bias was unlikely to be a major factor. Although the results should be interpreted with caution, the present study suggests that screening using immunochemical FOBT provides protection against development of advanced stage colorectal cancer.
